# Helix Electrohydrodynamic Printing of Highly Aligned Serpentine Micro/Nanofibers

**DOI:** 10.3390/polym9090434

**Published:** 2017-09-08

**Authors:** Yongqing Duan, Yajiang Ding, Zhoulong Xu, YongAn Huang, Zhouping Yin

**Affiliations:** 1 State Key Laboratory of Digital Manufacturing Equipment and Technology, Huazhong University of Science and Technology, Wuhan 430074, China; duanyongqing@hust.edu.cn (Y.D.); dyjqwert@gmail.com (Y.D.); xuzhoulong@hust.edu.cn (Z.X.); 2 Flexible Electronics Research Center, Huazhong University of Science and Technology, Wuhan 430074, China

**Keywords:** serpentine micro/nanofiber, electrospinning, stretchable electronics

## Abstract

Micro/nano serpentine structures have widespread applications in flexible/stretchable electronics; however, challenges still exist for low-cost, high-efficiency and controllable manufacturing. Helix electrohydrodynamic printing (HE-printing) has been proposed here to realize controllable direct-writing of large area, highly aligned serpentine micro/nanofibers by introducing the rope coiling effect into printing process. By manipulating the flying trajectory and solidification degree of the micro/nano jet, the solidified micro/nanofiber flying in a stabilized helical manner and versatile serpentine structures deposited on a moving collector have been achieved. Systematic experiments and theoretical analysis were conducted to study the transformation behavior and the size changing rules for various deposited microstructures, and highly aligned serpentine microfibers were directly written by controlling the applied voltage, nozzle-to-collector distance and collector velocity. Furthermore, a hyper-stretchable piezoelectric device that can detect stretching, bending and pressure has been successfully fabricated using the printed serpentine micro/nanofibers, demonstrating the potential of HE-printing in stretchable electronics manufacturing.

## 1. Introduction

The rapid development of stretchable electronics that can accommodate large deformation (>>1%) has accelerated a wide range of new applications, such as epidermal electronics [1,2,3], hemispherical bionic cameras [4], stretchable batteries [5,6] and implantable devices [7]. Of particular importance are the design and fabrication of stretchable architectures, such as buckled [8], serpentine [1,9], self-similar [5,10], helical [11] and textile structures [12,13], which enable devices to be stretched, compressed, twisted, etc. Serpentine structures widely exist in our daily life, such as plant tendrils and curled hair, and they have been recently considered as one of the dominating designs for stretchable architectures due to their excellent mechanical performance. Serpentine micro/nano structures are mostly fabricated through conventional micro/nano processing techniques, such as lithography, etching and sputtering, which require special equipment and complex processes with high cost and low efficiency. With the development of solution-processable functional materials, printing techniques become attractive in large area pattern deposition and flexible electronics fabrication. However, traditional drop-on-demand inkjet printing still faces intractable problems in stretchable micro/nano serpentine structures generation, i.e., printing resolution and pattern quality. Inkjet printing has difficulty depositing structures smaller than 20 μm; meanwhile, it is difficult to achieve continuous, uniform and smooth stretchable architectures through discontinuous drop-on-demand printing. How to fabricate micro/nano serpentine structures in a simpler, more cost-effective way is of significant importance.

Electrohydrodynamic printing, well known for its use in electrospinning and electrospray, has recently been employed in high resolution micro/nano fabrication. Electrospinning is a process that ejects continuous micro/nano jet from highly viscous fluid through imposed high electric force; it is a simple, cost-effective and high-efficient method for micro/nanofibers generation [14,15,16]. Electrospinning overcomes the limitation of printing resolution and discontinuous printing feature, it may be efficient in serpentine micro/nanofibers generation, however, difficulties exist in orientation and controllability. Traditional far-field electrospinning only generates disordered fibers or nonwoven mats due to the whipping/bending instability caused by electrical repulsive force [17]. By shortening the nozzle-to-collector distance, near-field electrospinning (NFES) can avoid the whipping instability and realize the direct-writing of straight micro/nano fibers [18]. This controllable manner has greatly expanded the applications of electrospinning, i.e., transparent electrodes [19], piezoelectric devices [20], non-volatile memories [21] and field-effect transistors [22]. However, the straight fiber based structures enable devices to be bendable but not stretchable.

To realize controllable manufacturing of stretchable helical/serpentine micro/nano fibers, several attempts have been proposed. For example, Duan et al. [23] achieved self-organized serpentine fibers by depositing straight fibers onto a prestrained elastomer, then releasing the prestrain of the elastomer. Fang et al. [24] utilized an auxiliary electrode worked under alternating current, making the jet swing back and forth to deposit serpentine fibers on collector. These assistant measures help the jet/fiber transit from straight line to curve, but inevitably bring new control problems. Buckling phenomenon of the electrospinning jet has long been reported having superior performance for serpentine fibers generation [25,26,27]. The electrospinning fiber buckles when striking on a collector caused by compression at impingement on a collector surface [28], which is similar to the rope coiling effect in nature that a thin viscous fluid or elastic rope coils based on mechanical buckling [29,30], but the relatively poor positioning and controllability in electrospinning process restrict its practical applications. To enhance controllability, Kim et al. [31] obtained regular coiling by directly electrospun polyethylene oxide solution over a static electrode tip instead of a planar electrode, the concentrated electric field distribution prevented the jet from uncontrollable whipping. Xin et al. [32] achieved regular hierarchical polystyrene patterns by depositing buckled jet over a moving collector. Although the relevant research has made some progress, challenges still exist in producing micro/nano serpentine structures with precise wavelength and amplitude.

In this paper, highly aligned serpentine polyvinylidine fluoride (PVDF) micro/nanofibers have been fabricated through a newly-proposed helix electrohydrodynamic printing (HE-printing) technique. By individually manipulating the solidified electrospun fiber flying in a stabilized helical manner as microscale elastic rope coiling, versatile serpentine structures can be direct-written on a moving collector over a wide range of scales. Experimental and theoretical studies have been conducted to analyze the effects of process parameters (e.g., applied voltage, nozzle-to-collector distance and collector velocity) on the pattern shape and reveal the transformation rules of different direct-written curve patterns. Meanwhile, a hyper-stretchable (>200%) piezoelectric device that can detect stretching, bending and pressure has been successfully fabricated, demonstrating the ultra stretchability of the printed serpentine PVDF micro/nanofibers and the enormous potential of HE-printing in stretchable electronics manufacturing.

## 2. Experimental Setup

### 2.1. Preparation of PVDF Solution and Elastomer Substrates

PVDF (Kynar 761, *M*_w_ = 440,000) was purchased from Arkema Investment Co. Ltd. (Paris, France), and N,N-dimethylformamide (DMF) and acetone were purchased from Sinopharm Chemical Reagent Co., Ltd. (Shanghai, China). A total of 1.8 g PVDF was added in the solvent mixture of 4.1 g DMF and 4.1 g acetone to obtain a concentration of 18%, and kept at 35 °C for about 6 h until the solution was homogeneous.

The Ecoflex substrate (Ecoflex 0030, Smooth-On Inc., Macungie, PA, USA) with microchannels was prepared by mixing the base and the curing agent with a ratio of 1:1, then the solution was placed in a vacuum oven to remove air bubbles, finally casted into a mold and thermally cured at 60 °C for 10 min. For the PDMS-on-Ecoflex substrate, Ecoflex solution was first spin-coated on a glass substrate with a thickness of about 0.3 mm, followed by thermal curing at 60 °C for 10 min. Then, PDMS solution (sylgard 184, Dow Corning, Inc., Midland, MI, USA) (mixing the base and the curing agent with a ratio of 10:1, and removing air bubbles by placing it in a vacuum oven) was spin-coated on the flat Ecoflex substrate, and thermally cured at 60 °C for 40 min to obtain a half cured PDMS layer with a thickness of about 0.1 mm.

### 2.2. HE-Printing of PVDF Micro/Nanofibers

**Figure 1:**
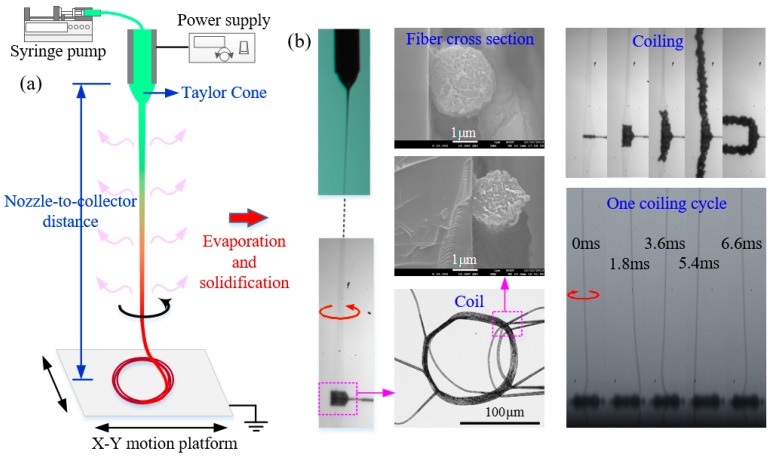
(**a**) Schematic diagram of HE-printing process; and (**b**) the left column represents the electrospun fiber flying in a helical manner, the middle column shows the coil fibers deposited on collector and their cross-sections, the right column demonstrates that the coil growth over time, and the coil can be over several millimeters until it collapses.

The schematic diagram of HE-printing was shown in Figure 1a. The PVDF solution was delivered using a syringe pump (11 Pico Plus, Harvard Apparatus, Holliston, MA, USA) at a feed rate of 400 nL/min. A high voltage was exerted between a metallic needle (inner diameter 260 μm and external diameter 510 μm) and a flat aluminum collector to generate Taylor cone and assist to pull out the jet through a current power supplier (DW-P403, Dongwen Inc., Tianjin, China). Three key process parameters were adjusted to get desired electrospun patterns: the applied voltage, the nozzle-to-collector distance and the collector speed. The applied voltage was set 1.5–3 kV. The nozzle-to-collector distance was varied from 10 to 50 mm by a manual slide. The collector is either motionless or moving horizontally; its moving speed was adjusted from 0 to 400 mm/min. The printing process was observed by a high speed camera (PCO.Dimax HD, PCO Inc., Lower Bavaria, Germany). The experiments were conducted at room temperature, 35–45% relatively humidity.

### 2.3. Device Fabrication

The direct-written serpentine micro/nanofibers were transferred onto a bidirectionally stretched PDMS-on-Ecoflex substrate by placing the bidirectionally stretched elastomer onto a silicon substrate with PVDF micro/nanofibers for several minutes, then self-similar micro/nanofibers were obtained by quickly peeling off the elastomer. 

The device was fabricated by bonding the patterned Ecoflex substrate onto the PDMS-on-Ecoflex substrate with PVDF micro/nanofibers to form microstructured channels, followed by injecting liquid metal (EgaIn, 75% gallium and 25% indium) to form stretchable interdigital electrodes.

### 2.4. Characterization

The viscosity of PVDF solution was measured by a viscometer (DV-I Prime, BrookField, Middleboro, MA, USA) at 25 °C. Morphological features of the electrospun micro/nanofibers were observed with a laser scanning confocal microscopy (LSCM, Keyence VK-X200K, Keyence, Osaka, Japan) and a Sirion 200 scanning electron microscope (SEM). All samples were coated with gold under vacuum before SEM test. The electric properties of the serpentine PVDF fibers were obtained through a semiconductor characterization system (Keithley 4200-SCS, Keithley, Cleveland, OH, USA) and a probe system (Cascade Summit 11000, Cascade, Beaverton, OR, USA).

## 3. Results and Discussion

### 3.1. HE-Printing Technique

HE-printing is capable of manipulating the fiber flying in a stabilized helical manner as shown in Figure 1a, completely different from disordered fiber of traditional far-field electrospinning and straight fiber of near-field electrospinning. The electrospinning jet is a continuous fluid flow ejected from the Taylor cone when the applied electrical force overcomes the surface tension of the hanging drop at the jetting nozzle. The jet moves straight away from the tip, then quickly downward pulled by the electrical force until it strikes onto the collector. Unlike near-field electrospinning, which has rather small nozzle-to-collector distance, HE-printing adopts relatively large nozzle-to-collector distance to realize tunable solidification of the electrospinning jet. Small nozzle-to-collector distance prefers liquid state for the jet when approaching collector, the liquid jet is accumulated into a liquid dot on a stationary collector and forms straight line via the linear movement of collector, otherwise, it may experience complex buckling of viscoelastic liquids and generate irregular winding structures on a moving collector. With the increase of nozzle-to-collector distance, the micro/nano liquid jet evaporates and solidifies, and finally the jet (when approaching collector) turns to be a solidified fiber with nearly circular cross-section (Figure 1b). The solidified fiber firstly buckles as it undergoes a compressive force at impingement on a collector surface, then it rapidly rotates and piles up on the collector as a spring coil (Figure 1b). The micro/nanofiber in HE-printing consists of two distinct parts: a long, roughly vertical “tail” (with length in millimeters to centimeters, and diameter in micrometers to nanometers) which deforms primarily by severe electrical stretching, and a helical “coil” in which the deformation is dominated by bending and twisting. To ensure enough time for the jet to solidify in air, and prevent the long, straight “tail” from disordered electrical whipping, several methods can be adopted: (a) carefully increasing the nozzle-to-collector distance while decreasing the applied voltage; (b) accelerating the evaporation behavior by controlling the environment or modifying the solution parameters (e.g., concentration and volatility); and (c) adopting an electrode ring around the jet or a sharp needle electrode underneath the plate electrode to regulate electric field distribution and prevent the jet from whipping. When the applied voltage was between 1.5 and 3 kV, the nozzle-to-collector distance varied from 10 to 50 mm, the experiments were conducted at room temperature, 35–45% relatively humidity, the electrospun PVDF jet (18 wt %, with viscosity of about 1900 mPa⋅s) transitioned to a solidified fiber and formed regular buckling coils when landing on collector, with coiling diameter, coiling cycle and fiber diameter of about a few hundred micrometers (30–300 μm), several milliseconds (1–10 ms) and several micrometers (1.5–3 μm), respectively.

**Figure 2:**
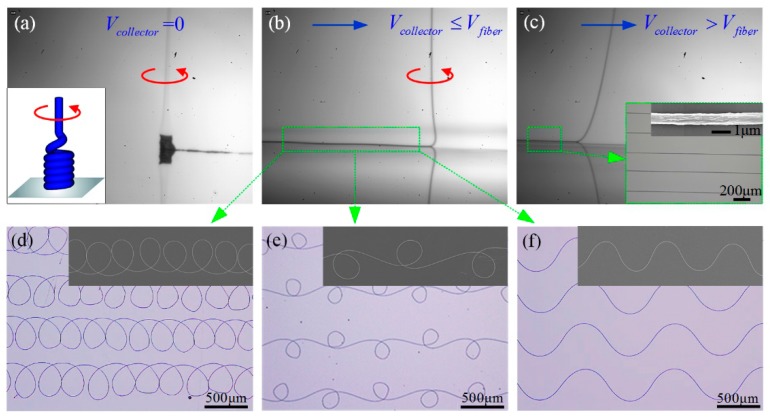
(**a**–**c**) The electrospun jet exhibits a spring-like structure, a buckled heel or a stretched catenary under different conditions. (**d**–**f**) The optical and SEM images of fibers deposited on collector with different applied voltages, nozzle to collector distances and collector velocities. The parameters are: (**d**) 2.1 kV, 2.5 cm, and 100 mm/s; (**e**) 2.1 kV, 2.5 cm, and 150 mm/s; and (**f**) 2.1 kV, 2.5 cm, and 200 mm/s.

The helical fiber is accumulated into a spring-like structure on a stationary collector, and forms versatile structures when collector moves with different speeds, as shown in Figure 2. Once the coiled fiber is dispersed along the moving direction, patterns from curve to straight can be formed on collector. The lowermost part of the filament can exhibit three distinct dynamical regimes depending on different conditions, that is whether the collector speed *V*_collector_ is greater than, nearly equal to, or less than the critical downward speed of the fiber when approaching collector *V*_fiber_. (1) *V*_collector_ > *V*_fiber_ means that the jet length carried away by collector is larger than the jet length falls on collector per unit time. The stretched jet is a steady dragged catenary, and oriented straight fibers are generated [33,34] (Figure 2c). (2) *V*_collector_ ≈ *V*_fiber_ means that, as the collector speed is decreased towards *V*_fiber_, the lowermost part of the filament evolves into a compressed “heel” shape (Figure 2b), which becomes unstable to periodic meandering. (3) *V*_collector_ < *V*_fiber_ means that further decrease of the collector velocity leads to a series of bifurcations, and three dominant patterns are meandering, alternating loops and translated coiling (Figure 2d–f), ending with the static coiling on a stationary collector (Figure 2a).

By adjusting process parameters, e.g., applied voltage *U*_applied_, nozzle-to-collector distance *D*_collector_ and collector speed *V*_collector_, the helix motion of the electrical-filed-driven jet can be controlled, and positioned translated coiling, alternating loops and meandering patterns can be deposited onto collector. To realize direct-writing of serpentine microstructures, the influences of process parameters and the transformation rules between different patterns should be clarified.

### 3.2. Influences of Process Parameters

**Figure 3:**
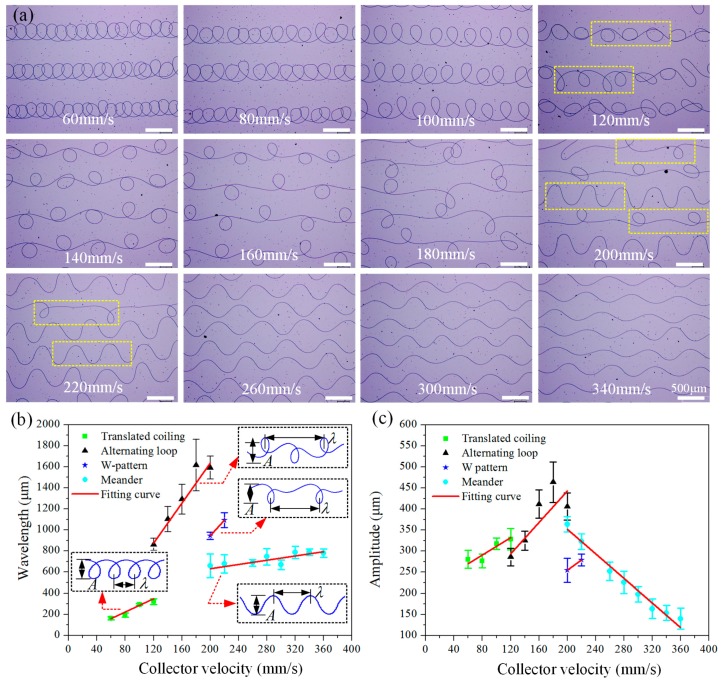
(**a**) Patterns deposited under different collector velocities. (**b**,**c**) The wavelength (**b**); and amplitude (**c**) of different electrospun patterns vary over collector velocity. The wavelength and amplitude of each pattern are defined in the exerted images of (**b**). (The scale bars in Figure 3a are 500 μm).

Figure 3a shows the patterns deposited on collector when gradually increasing the collector speed, at the case of *D*_collector_ = 2.5 cm and *U*_applied_ = 2.2 kV. When *V*_collector_ < 100 mm/s, only translated coiling appears, and the patterns become loose with the increase of collector speed. When *V*_collector_ is about 120 mm/s, translated coiling becomes unstable and begins to transit to alternating loops, and both patterns coexist at this moment. With further increase of *V*_collector_ from 140 to 180 mm/s, alternating loops are dominant and the pattern becomes increasingly elongated. When *V*_collector_ is about 200–220 mm/s, alternating loops lose their stability and begin to transit to meanders. Meanwhile, a new W-shaped pattern appears in this region, and the three patterns, alternating loops, W pattern and meander, may coexist. After that, only meanders appear. When *V*_collector_ is larger than 360 mm/s, the configuration of the hanging fiber changes from a “heel” shape (Figure 2b) to a stretched catenary (Figure 2c), and the deposited pattern eventually turns into a straight line (Figure 2c).

Figure 3b,c summarizes the change rules of wavelength and amplitude of deposited patterns over collector speed, where wavelength and amplitude of each pattern are defined in the exerted images of Figure 3b. With the increase of collector velocity, wavelength of each pattern has an increasing trend, while the variation of amplitude is inconsistent. Translated coiling, alternating loops and W-shaped pattern all have an increasing trend in amplitude, while meander has a decreasing trend as its critical curve is straight line. It should be noted that the discontinuous of wavelength in the pattern transformation region is mainly caused by its definition. For example, continuous wavelength can be obtained by redefining the wavelength of the alternating loops to be the distance between two loops, namely half of the existing definition.

**Figure 4:**
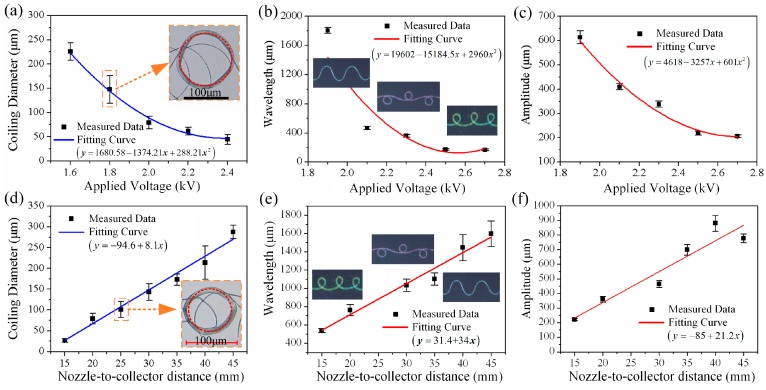
(**a**) The coiling diameter versus applied voltage when *D*_collector_ = 25 mm and *V*_collector_ = 0 mm/s. (**b**,**c**) The wavelength and amplitude of different patterns versus applied voltage when *D*_collector_ = 25 mm and *V*_collector_ = 200 mm/s. (**d**) The coiling diameter versus nozzle-to-collector distance when *U*_applied_ = 2.1 kV and *V*_collector_ = 0 mm/s. (**e**,**f**) The wavelength and amplitude of different patterns versus nozzle-to-collector distance when *U*_applied_ = 2.1 kV and *V*_collector_ = 200 mm/s. The formulas in parentheses are the fitting equations of each fitted curve.

Figure 4 illustrates the influences of nozzle-to-collector distance *D*_collector_ and applied voltage *U*_applied_ on the helix behavior and the deposition patterns. The coiling diameter decreases rapidly as *U*_applied_ increases when *D*_collector_ = 25 mm and *V*_collector_ = 0 (Figure 4a). Higher voltage leads to larger stretch force exerted on jet, thus larger jet velocity and smaller jet diameter, which finally induce a smaller coiling diameter. Figure 4b,c demonstrates that both geometric shape and size change with *U*_applied_ at the case of *D*_collector_ = 25 mm and *V*_collector_ = 200 mm/s. When increasing the applied voltage, the deposited fibers vary from straight to meander, alternating loops and finally translated coiling. Meanwhile, the pattern size decreases rapidly, which has a similar trend in Figure 4a. Figure 4d shows that the coiling diameter increases linearly with *D*_collector_ in the case of *U*_applied_ = 2.1 kV and *V*_collector_ = 0. Higher nozzle-to-collector distance corresponds to weaker electric filed, thus smaller critical velocity of the fiber *V*_fiber_ when striking on collector, and finally a larger coiling diameter. With the increase of nozzle-to-collector distance, the deposited fibers vary from translated coiling to alternating loops and meander when *U*_applied_ = 2.1 kV and *V*_collector_ = 200 mm/s (Figure 4e,f). Meanwhile, the pattern size increases linearly, which has a similar trend in Figure 4d. These may give a conclusion that the coiling diameter decided by applied voltage and nozzle-to-collector distance on a motionless collector largely determines the size of the complex patterns deposited on a moving collector.

### 3.3. Transformation Rules between Different Patterns

To reveal the transformation rules between different patterns, we first go to the similar buckling patterns of the gravity driven jets or ropes. When a thin viscous thread or elastic rope impinges on a collector, coiling happens on a stationary collector, and similar buckling patterns including translated coiling, alternating loops and meanders appear on a moving collector. This phenomenon is called the fluid mechanical sewing machine [35] or elastic sewing machine [36], which has inspired several experimental, theoretical and numerical works to investigate the essence of pattern transformation. The coiling behavior of the electrospun micro/nanofiber is similar to the gravity driven rope coiling, although their characteristic lengths are micro/nanoscale and centimeters/millimeters, respectively. Recently, Brun et al. [37,38] developed a quasi-static geometrical model (GM) to capture the various buckling patterns of thin viscous jet or elastic rope, and successfully calculated the bifurcation threshold of different patterns. They found that the jet/collector velocity match coefficient was the key factor for pattern variation, and this was coincidence with the HE-printing results.

**Figure 5:**
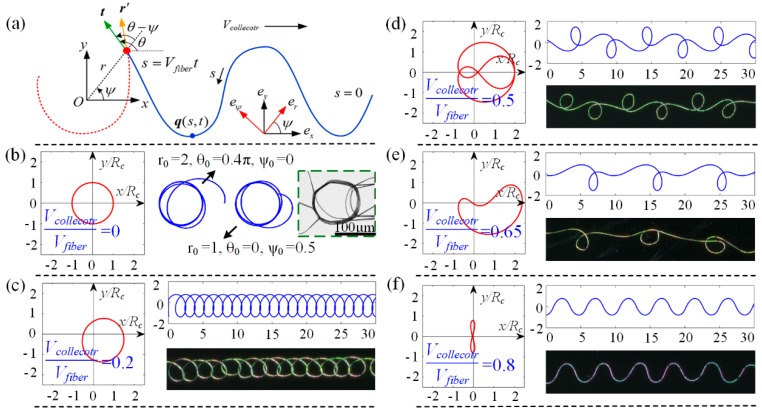
(**a**) Schematic diagram of the deposited trace q(s,t) and orbit of the contact point r(s) in the geometrical model. (**b**) The analytical and experimental results of regular coiling when collector is stationary. The orbit of the contact point r(s) converges to regular coiling whatever the initial values of $r_{0}$, $\psi_{0}$ and $\theta_{0}$ are. (**c**–**f**) The orbit of the contact point, the deposited trace and the printed patterns with different $V_{\mathrm{collector}}/V_{\mathrm{fiber}}$. (**c**–**f**) The velocity match coefficients $V_{\mathrm{collector}}/V_{\mathrm{fiber}}$ are 0.2, 0.5, 0.65, and 0.8, respectively.

In the geometrical model [37,38], the deposited trace on collector, namely the patterns of meander, alternating loops, translated coiling, etc. is a combination of the orbit of the contact point (regular coiling when collector is motionless) and the movement of the collector. The deposited trace q(s,t) in Figure 5a can be written as $q(s,t)=r(s)+V_{\mathrm{collector}}(t-s/V_{\mathrm{fiber}})e_{x}$, where *s* is the arc-length, *t* is time, r(s) means the contact point at time $s/V_{\mathrm{fiber}}$, $V_{\mathrm{fiber}}$ is the fiber velocity, *V*_collector_ and $e_{x}$ are the value and direction of the collector speed, and $t-s/V_{\mathrm{fiber}}$ means the time that the contact point move together with collector. From derivate q(s,t) with arc-length *s*, we can get $t(s)=\partial q(s,t)/\partial s{|}_{s=U_{c}t}=r^{\prime }(s)-V_{\mathrm{collector}}/V_{\mathrm{fiber}}e_{x}$, where t(s) and $r^{\prime }(s)$ are the tangent vector of q(s,t) and r(s), respectively. Next, we rewrite t(s) from a Cartesian coordinate $\left(e_{x},e_{y}\right)$ to a polar coordinate $\left(e_{r},e_{\psi }\right)$, while consider the curvature θ' at the bottom of the hanging thread that $\;\theta \;\prime =\frac{1}{R_{c}}\sqrt{\frac{r}{R_{c}}}\left(1+\frac{0.715^{2}\cos(\theta -\psi )}{1-0.715\cos(\theta -\psi )}\frac{r}{R_{c}}\right)\sin(\theta -\psi )$ [37], and remove the coiling radius $R_{c}$ by rescaling, to get

(1) $$\begin{array}{l} r^{\prime }=\cos(\theta -\psi )+\frac{V_{\mathrm{collector}}}{V_{\mathrm{fiber}}}\cos\psi \\ \;\psi \;\prime =\frac{1}{r}\left(\sin(\theta -\psi )-\frac{V_{\mathrm{collector}}}{V_{\mathrm{fiber}}}\sin\psi \right)\\ \;\theta \;\prime =\sqrt{r}\left(1+\frac{0.715^{2}\cos(\theta -\psi )}{1-0.715\cos(\theta -\psi )}r\right)\sin(\theta -\psi ) \end{array}$$

Equation (1) consists of a set of coupled ordinary nonlinear differential equations for the functions of r(s), ψ(s) and θ(s), and a dimensionless parameter $V_{\mathrm{collector}}/V_{\mathrm{fiber}}$. When varying $V_{\mathrm{collector}}/V_{\mathrm{fiber}}$($0\leq V_{\mathrm{collector}}/V_{\mathrm{fiber}}\leq 1$), the solutions of r(s), ψ(s) and θ(s), the deposited trace q(s,t) and the orbit of the contact point r(s) can be achieved.

When the collector is static, namely $V_{\mathrm{collector}}/V_{\mathrm{fiber}}=0$, the orbit of the contact point converges to a regular circle with a diameter of $2R_{c}$ (Figure 5b), and the calculation result is insensitive to the initial values of $r_{0}$, $\psi_{0}$ and $\theta_{0}$. The coordinates $x/R_{c}$ and $y/R_{c}$ are dimensionless, where $R_{c}$ means the regular coiling radius. Continuously increasing $V_{\mathrm{collector}}/V_{\mathrm{fiber}}$, translated coiling, alternating loops, W pattern and meander appear in succession; the four typical periodic orbits and corresponding patterns are listed in Figure 5c–f when the velocity match coefficients are 0.2, 0.5, 0.65, and 0.8, respectively. The analytical deposited traces are quite consistent with the experimental electrospun patterns. 

**Figure 6:**
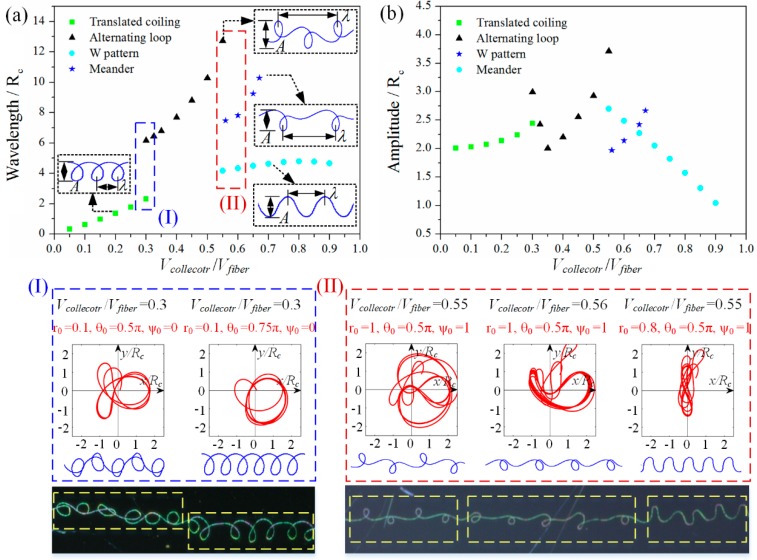
Analytical results of the: dimensionless wavelength (**a**); and amplitude (**b**) of different buckling patterns varies over velocity match ratio. Green rectangles, black triangles, blue stars and cyan circles represent translated coiling, alternating loop, W-pattern and meander, respectively. (**I**) The orbit of the contact point and the deposited trace calculated with different initial values of $r_{0}$, $\psi_{0}$, $\theta_{0}$ when $V_{\mathrm{collector}}/V_{\mathrm{fiber}}=0.3$. The bottom image shows one electrospun fiber with alternating loops and translated coiling simultaneously. (**II**) The orbit of the contact point and the deposited trace calculated with different initial values of $r_{0}$, $\psi_{0}$ and $\theta_{0}$ when $V_{\mathrm{collector}}/V_{\mathrm{fiber}}$ equals to 0.55 or 0.56. The bottom image shows one electrospun fiber with translated coiling, W pattern and meander simultaneously.

To clarify the transformation behavior and the size changing rules for different patterns, the deposited traces have been calculated under different $V_{\mathrm{collector}}/V_{\mathrm{fiber}}$ with different initial values. Figure 6a,b presents the dimensionless wavelength $\lambda /R_{c}$ and amplitude $A/R_{c}$ obtained from Equation (1), in which the dimensionless velocity $V_{\mathrm{collector}}/V_{\mathrm{fiber}}$ is abscissa; the wavelength λ and amplitude *A* of each pattern are defined in the exerted images of Figure 6a. The analytical results agree well with the HE-printing experiments (Figure 3), and we can conclude that: (1) The pattern type is determined by the velocity match coefficient $V_{\mathrm{collector}}/V_{\mathrm{fiber}}$. Four typical patterns appear in the correct order when $V_{\mathrm{collector}}/V_{\mathrm{fiber}}$ varies; however, only three patterns can be generated separately, as W pattern usually exists concomitantly in the pattern transition region. (2) The pattern size is determined by the regular coiling diameter $2R_{c}$, which has been validated through Figure 4. The size changing rules for different patterns in Figure 6a,b are quite coincident with experimental results (Figure 3b,c). Wavelength of each pattern increases with $V_{\mathrm{collector}}/V_{\mathrm{fiber}}$, while the amplitude has divergent trends. (3) The velocity match coefficient $V_{\mathrm{collector}}/V_{\mathrm{fiber}}$ and the regular coiling diameter $2R_{c}$ decide the direct-written pattern, which are influenced by three key parameters: the applied voltage *U*_applied_ and nozzle-to-collector distance *D*_collector_ decide the size of the coiling diameter $2R_{c}$ and the value of the critical fiber velocity when attaching collector $V_{\mathrm{fiber}}$. The collector velocity $V_{\mathrm{collector}}$ and the critical velocity $V_{\mathrm{fiber}}$ compose the velocity match coefficient $V_{\mathrm{collector}}/V_{\mathrm{fiber}}$. (4) The pattern transition regions (I) and (II) should be avoided in manufacturing process. When $V_{\mathrm{collector}}/V_{\mathrm{fiber}}$ is around 0.3, the deposited trace is unstable, meaning both coiling and alternating loops may appear under different initial values, in line with experimental results that two patterns may coexist in one fiber (region (I) in Figure 6). The transition from alternating loops to serpentine is complicated when $V_{\mathrm{collector}}/V_{\mathrm{fiber}}$ is around 0.55–0.67 (region (II) in Figure 6). Both analytical and experimental results prove that, whether alternating loops or serpentine, W pattern occurs simultaneously in this region, and the three patterns can exist in one electrospun fiber.

### 3.4. Potential Applications

**Figure 7:**
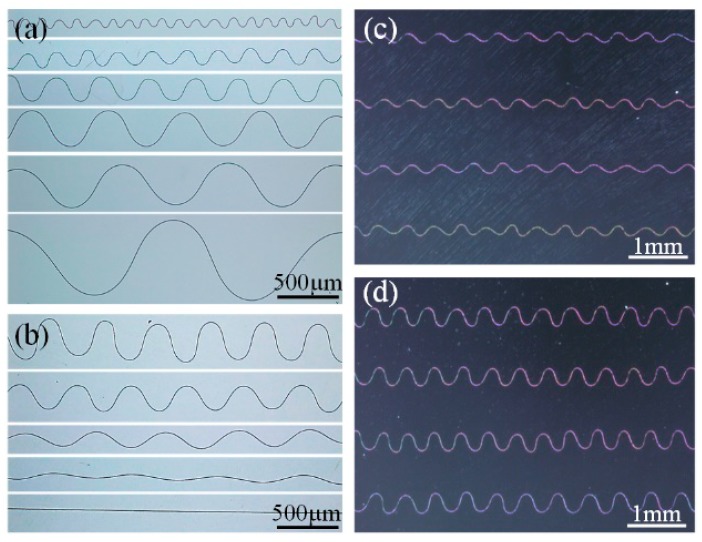
(**a**,**b**) Serpentine micro/nanofibers with different sizes and wave forms; and (**c**,**d**) highly aligned, large area serpentine micro/nanofibers.

As discussed above, HE-printing is an efficient method for stretchable serpentine structure fabrication. By regulating three key process parameters (*U*_applied_, *D*_collector_ and *V*_collector_) to tune the velocity match coefficient $V_{\mathrm{collector}}/V_{\mathrm{fiber}}$ and the regular coiling diameter $2R_{c}$, HE-printing is capable of depositing serpentine structures with specific wavelength and amplitude, as shown in Figure 7a,b. The wavelength range varies from about 100 to 2000 μm using the process parameters: *U*_applied_ = 1.5–3 kV, *D*_collector_ = 10–50 mm, and *V*_collector_ = 0–400 mm/min. The ratio of wavelength to amplitude varies from about 1.4 to infinity, where 1.4 corresponds to the transition point that the serpentine structures bifurcate into alternating loops or W-shaped pattern, and infinity corresponds to the limit state of straight line. The serpentine structures are reproducible, and they can be generated in a large scale, as shown in Figure 7c,d.

In addition to serpentine structures, a more complex self-similar structure has been recently considered as an important approach to achieve very large stretchability (e.g., > 100%) as it adopts spring-on-spring architecture, and it has been used to enhance the stretchability of lithium ion batteries up to 300% [5]. The complex self-similar structures are mainly fabricated through photolithography and etching, which are either complicated and expensive or incompatible with large-scale, low-cost fabrication.

**Figure 8:**
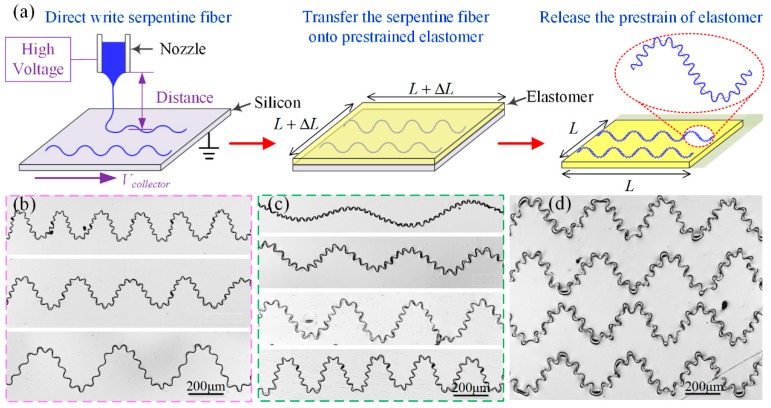
(**a**) Schematic diagram of the fabrication process of self-similar serpentine micro/nanofibers. (**b**,**c**) Self-similar serpentine micro/nanofibers with different: sizes (**b**); and wave forms (**c**). (**d**) Highly aligned, large area self-similar serpentine micro/nanofibers.

Here, a new two-level wave-shaped self-similar structure has been fabricated by combination of HE-printing and self-organized buckling. Figure 8a illustrates the three-step process: (1) Direct-write the initial serpentine micro/nanofibers on a silicon substrate through HE-Printing, with tunable wavelength and amplitude, as shown in Figure 7. (2) Transfer the initial serpentine micro/nanofibers to a biaxially prestrained PDMS-on-Ecoflex substrate by pressing the prestrained elastomer on the silicon wafer with serpentine micro/nanofibers. PDMS-on-Ecoflex substrate is utilized here considering the surface stickiness of fresh PDMS to achieve high bonding strength with serpentine micro/nanofibers, and the hyperelasticity of Ecoflex to improve stretchability. (3) Peel off and release the biaxial prestrain of elastomer to cause self-organized buckling of serpentine micro/nanofibers into self-similar fibers. When releasing the biaxial prestrain of the elastomer, the initial serpentine geometry (λ_initial_ and *A*_initial_) proportionally shrinks to the first-level wavy geometry (λ_level1_ and *A*_level1_), while buckling to the second-level wavy geometry (λ_level2_ and *A*_level2_), namely λ_level1_ = λ_initial_/(1 + ε) and *A*_level1_ = *A*_initial_/(1 + ε), where ε is the biaxial prestrain of elastomer. λ_level2_ and *A*_level2_ are controlled by the prestrain of elastomer, Young’s modulus and cross-sectional geometry of fiber and substrate basing on buckling mechanics, and the serpentine fibers buckle in-plane as the fiber cross-section is nearly circular. Figure 8b–d demonstrates self-similar micro/nanofibers with various sizes, various aspect ratios or in large area and self-organized (Figure 7), and these conformal self-similar fibers may have great potential in stretchable electronics.

**Figure 9:**
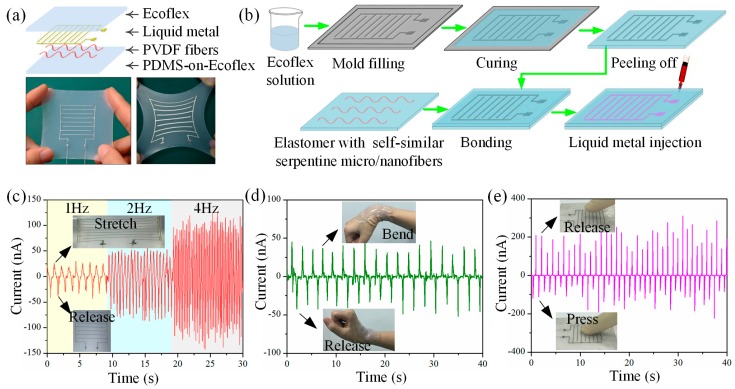
(**a**) Schematic diagram of the hyper-stretchable piezoelectric device in a layer by layer format and optical images of the device in initial and stretched status. (**b**) Schematic diagram of the fabrication process of the stretchable piezoelectric device. (**c**–**e**) Current outputs of the hyper-stretchable piezoelectric device subjected to stretch (200%) and release with different frequencies (**c**); bending to monitor the movement of human wrist (**d**); and pressure with human fingers (**e**).

Considering that the electrospun PVDF fibers have excellent piezoelectricity [20], a hyper-stretchable piezoelectric device based on printed serpentine micro/nanofibers has been fabricated and tested. The schematic diagram of the hyper-stretchable piezoelectric device in a layer by layer format is shown in Figure 9a. The sandwich-structured composite consists of four layers: a PVDF layer for electricity generation, a liquid metal layer for charge transfer, and two elastomer layers for encapsulation. The concrete fabrication process is shown in Figure 9b. Ecoflex substrate with microchannels was bonded onto the flat PDMS-on-Ecoflex substrate with PVDF micro/nanofibers to form microstructured channels, followed by injecting liquid metal to form stretchable interdigital electrodes. Self-similar serpentine PVDF fibers, liquid metal electrodes, and Ecoflex encapsulations enhance the stretchability of the device which is able to undergo large stretchability without failure. Figure 9c shows the output current of the device (with about 150 serpentine PVDF fibers) characterized by a semiconductor characterization system under an applied strain of 200% at different stretch and release frequencies. The stable output under large deformation demonstrates the stretchability of the printed serpentine fibers. Meanwhile, there is a linear relationship between the average maximum current and the reciprocating frequency, which is consistent with the fundamental piezoelectric theory that the generated current is proportional to the strain rate. Figure 9d,e illustrates that the device is also sensitive to bending and pressure, which has potential artificial skin applications for monitoring human movement and external stimuli.

## 4. Conclusions

HE-printing has been proposed to realize controllable fabrication of highly oriented helical/serpentine micro/nanofibers. Regular coiling on a stationary collector and a variety of reproducible patterns on a moving collector were achieved by ensuring that the micro/nano jet totally solidifies in air, while stabilizing it from uncontrollable electrical whipping instability. The reproducible electrospun patterns are almost identical in the gravity driven rope coiling, which are determined by the velocity match coefficient $V_{\mathrm{collector}}/V_{\mathrm{fiber}}$ and the regular coiling diameter $2R_{c}$. The former is mainly manipulated by varying collector velocity, and the latter decreases with applied voltage and linearly increases with nozzle-to-collector distance. The direct-written serpentine micro/nanofibers can be further self-organized into self-similar serpentine structures, and employed into hyper-stretchable and ultra-stable piezoelectric devices, showing great potential applications in stretchable sensors and artificial skins.

## References

[1] Yeo W.H., Kim Y.S., Lee J., Ameen A., Shi L., Li M., Wang S., Ma R., Jin S.H., Kang Z. (2013). Multifunctional epidermal electronics printed directly onto the skin. Adv. Mater..

[2] He X., Zi Y., Guo H., Zheng H., Xi Y., Wu C., Wang J., Zhang W., Lu C., Wang Z.L. (2017). A highly stretchable fiber-based triboelectric nanogenerator for self-powered wearable electronics. Adv. Funct. Mater..

[3] Kim J., Banks A., Cheng H., Xie Z., Xu S., Jang K.I., Lee J.W., Liu Z., Gutruf P., Huang X. (2015). Epidermal electronics with advanced capabilities in near-field communication. Small.

[4] Ko H.C., Stoykovich M.P., Song J., Malyarchuk V., Choi W.M., Yu C.-J., Geddes Iii J.B., Xiao J., Wang S., Huang Y. (2008). A hemispherical electronic eye camera based on compressible silicon optoelectronics. Nature.

[5] Xu S., Zhang Y., Cho J., Lee J., Huang X., Jia L., Fan J.A., Su Y., Su J., Zhang H. (2013). Stretchable batteries with self-similar serpentine interconnects and integrated wireless recharging systems. Nat. Commun..

[6] Kumar R., Shin J., Yin L., You J.M., Meng Y.S., Wang J. (2017). All-printed, stretchable Zn-Ag2O rechargeable battery via hyperelastic binder for self-powering wearable electronics. Adv. Energy Mater..

[7] Park S.I., Brenner D.S., Shin G., Morgan C.D., Copits B.A., Chung H.U., Pullen M.Y., Noh K.N., Davidson S., Oh S.J. (2015). Soft, stretchable, fully implantable miniaturized optoelectronic systems for wireless optogenetics. Nat. Biotechnol..

[8] Khang D.-Y., Jiang H., Huang Y., Rogers J.A. (2006). A stretchable form of single-crystal silicon for high-performance electronics on rubber substrates. Science.

[9] Tian L., Li Y., Webb R.C., Krishnan S., Bian Z., Song J., Ning X., Crawford K., Kurniawan J., Bonifas A. (2017). Flexible and stretchable 3ω sensors for thermal characterization of human skin. Adv. Funct. Mater..

[10] Fan J.A., Yeo W.-H., Su Y., Hattori Y., Lee W., Jung S.-Y., Zhang Y., Liu Z., Cheng H., Falgout L. (2014). Fractal design concepts for stretchable electronics. Nat. Commun..

[11] Won Y., Kim A., Yang W., Jeong S., Moon J. (2014). A highly stretchable, helical copper nanowire conductor exhibiting a stretchability of 700%. NPG Asia Mater..

[12] Hu L., Pasta M., Mantia F.L., Cui L., Jeong S., Deshazer H.D., Choi J.W., Han S.M., Cui Y. (2010). Stretchable, porous, and conductive energy textiles. Nano Lett..

[13] Stoppa M., Chiolerio A. (2014). Wearable Electronics and Smart Textiles: A Critical Review. Sensors.

[14] Lee Y.-S., Livingston Arinzeh T. (2011). Electrospun nanofibrous materials for neural tissue engineering. Polymers.

[15] Persano L., Camposeo A., Tekmen C., Pisignano D. (2013). Industrial upscaling of electrospinning and applications of polymer nanofibers: A review. Macromol. Mater. Eng..

[16] Sun B., Long Y.-Z., Chen Z.-J., Liu S.-L., Zhang H.-D., Zhang J.-C., Han W.-P. (2014). Recent advances in flexible and stretchable electronic devices via electrospinning. J. Mater. Chem. C.

[17] Reneker D.H., Yarin A.L., Fong H., Koombhongse S. (2000). Bending instability of electrically charged liquid jets of polymer solutions in electrospinning. J. Appl. Phys..

[18] Sun D., Chang C., Li S., Lin L. (2006). Near-field electrospinning. Nano Lett..

[19] Lee Y., Min S.Y., Kim T.S., Jeong S.H., Won J.Y., Kim H., Xu W., Jeong J.K., Lee T.W. (2016). Versatile metal nanowiring platform for large-scale nano-and opto-electronic devices. Adv. Mater..

[20] Chang C., Tran V.H., Wang J., Fuh Y.-K., Lin L. (2010). Direct-write piezoelectric polymeric nanogenerator with high energy conversion efficiency. Nano Lett..

[21] Hwang S.K., Min S.Y., Bae I., Cho S.M., Kim K.L., Lee T.W., Park C. (2014). Non-volatile ferroelectric memory with position-addressable polymer semiconducting nanowire. Small.

[22] Min S.-Y., Kim T.-S., Kim B.J., Cho H., Noh Y.-Y., Yang H., Cho J.H., Lee T.-W. (2013). Large-scale organic nanowire lithography and electronics. Nat. Commun..

[23] Duan Y., Huang Y., Yin Z., Bu N., Dong W. (2014). Non-wrinkled, highly stretchable piezoelectric devices by electrohydrodynamic direct-writing. Nanoscale.

[24] Fang F., Chen X., Du Z., Zhu Z., Chen X., Wang H., Wu P. (2015). Controllable direct-writing of serpentine micro/nano structures via low voltage electrospinning. Polymers.

[25] Yu J., Qiu Y., Zha X., Yu M., Yu J., Rafique J., Yin J. (2008). Production of aligned helical polymer nanofibers by electrospinning. Eur. Polym. J..

[26] Yao Y., Yin W., Cao J., Yang M., Li J., Zhao S., Li Y., He X., Leng J. (2014). Manipulation and formation mechanism of silica one-dimensional periodic structures by roller electrospinning. Langmuir.

[27] Shariatpanahi S., Abdollahzadeh I., Shirsavar R., Bonn D., Ejtehadi R. (2011). Micro helical polymeric structures produced by variable voltage direct electrospinning. Soft Matter.

[28] Han T., Reneker D.H., Yarin A.L. (2007). Buckling of jets in electrospinning. Polymer.

[29] Ribe N.M., Habibi M., Bonn D. (2012). Liquid rope coiling. Annu. Rev. Fluid Mech..

[30] Habibi M., Ribe N., Bonn D. (2007). Coiling of elastic ropes. Phys. Rev. Lett..

[31] Kim H.-Y., Lee M., Park K.J., Kim S., Mahadevan L. (2010). Nanopottery: Coiling of electrospun polymer nanofibers. Nano Lett..

[32] Xin Y., Reneker D.H. (2012). Hierarchical polystyrene patterns produced by electrospinning. Polymer.

[33] Huang Y., Wang X., Duan Y., Bu N., Yin Z. (2012). Controllable self-organization of colloid microarrays based on finite length effects of electrospun ribbons. Soft Matter.

[34] Bu N., Huang Y., Duan Y., Ding Y., Yin Z. (2015). Near-field behavior of electrified jet under moving substrate constrains. AIP Adv..

[35] Chiu-Webster S., Lister J. (2006). The fall of a viscous thread onto a moving surface: A ‘fluid-mechanical sewing machine’. J. Fluid Mech..

[36] Habibi M., Najafi J., Ribe N.M. (2011). Pattern formation in a thread falling onto a moving belt: An “elastic sewing machine”. Phys. Rev. E.

[37] Brun P.-T., Audoly B., Ribe N.M., Eaves T.S., Lister J.R. (2015). Liquid ropes: A geometrical model for thin viscous jet instabilities. Phys. Rev. Lett..

[38] Jawed M.K., Brun P.-T., Reis P.M. (2015). A geometric model for the coiling of an elastic rod deployed onto a moving substrate. J. Appl. Mech..

